# Comparative Study of Aluminum-Doped Zinc Oxide, Gallium-Doped Zinc Oxide and Indium-Doped Tin Oxide Thin Films Deposited by Radio Frequency Magnetron Sputtering

**DOI:** 10.3390/nano12091539

**Published:** 2022-05-02

**Authors:** Shadab Khan, Eugen Stamate

**Affiliations:** National Center for Nano Fabrication and Characterization, Technical University of Denmark, Ørsteds Plads 347, 2800 Kongens Lyngby, Denmark

**Keywords:** transparent conducting oxides, aluminum-doped zinc oxide, gallium-doped zinc oxide, indium-doped tin oxide, metal oxides, magnetron sputtering

## Abstract

A timely replacement of the rather expensive indium-doped tin oxide with aluminum-doped zinc oxide is hindered by the poor uniformity of electronic properties when deposited by magnetron sputtering. Recent results demonstrated the ability to improve the uniformity and to decrease the resistivity of aluminum-doped zinc oxide thin films by decreasing the energy of the oxygen-negative ions assisting in thin film growth by using a tuning electrode. In this context, a comparative study was designed to elucidate if the same phenomenology holds for gallium-doped zinc oxide and indium-doped tin oxide as well. The metal oxide thin films have been deposited in the same setup for similar discharge parameters, and their properties were measured with high spatial resolution and correlated with the erosion track on the target’s surface. Furthermore, the films were also subject to post annealing and degradation tests by wet etching. While the tuning electrode was able to reduce the self-bias for all three materials, only the doped zinc oxide films exhibited properties correlating with the erosion track.

## 1. Introduction

Transparent conducting oxide (TCO) thin films [1,2,3] are essential for solar cells [4,5], touch panels [6], smart windows [7] and organic light emitting diodes [8]. The already tremendous surface area coated by TCO is expected to further increase due to the stringent need for efficient energy harvesting devices and accelerated implementation of energy-saving systems [9,10]. Moreover, this extensive use comes with high demands on raw material availability, low fabrication cost and thin film deposition methods compatible with a large area production [1]. While several techniques are available for TCO deposition, including chemical methods, spray pyrolysis and spin coating [2], magnetron sputtering is widely used due to its easy process integration with the subsequent addition of functional layers [2]. So far, indium tin oxide (ITO), deposited by magnetron sputtering, is the best available material due to its resistivity being below 2 × 10^−4^ Ω cm and average transmittance (400 to 700 nm) being above 80% [11]. However, the 10% doping level with indium is rather high when taking into consideration that indium is almost as available as silver and that it is also extensively used for soldering, alloying and other applications. To address this issue, alternative TCO materials have been under investigation for more than three decades, with aluminum-doped zinc oxide (AZO) as one of the most promising possibilities, considering the large availability of Al and Zn [12]. In the context of trying to achieve optoelectronic properties comparable to ITO it was realized that AZO thin films deposited by magnetron sputtering are highly nonuniform, with record low resistivities below 5 × 10^−4^ Ω cm obtained only for very small areas, usually near the edge of the substrate [3,13]. The intensive research dedicated to understanding the reason behind this observation pointed to the possible role of energetic oxygen negative ions, which assist film growth [14,15,16,17,18,19,20,21,22,23,24,25]. These negative ions result from atomic oxygen’s ability to attach low-energy electrons released by secondary emissions at target surfaces, where they see an acceleration potential over the space charge adjacent to the target. Depending on the operation mode of the magnetron sputtering discharge (DC or RF), it was demonstrated that the oxygen negative ion energy can exceed a few hundred eV [26]. Moreover, it was possible to identify a direct correlation between the erosion track on the target and the spatial distribution of the resistivity over the substrate [26]. By measuring the AZO thin film properties (sheet resistance, film thickness, band gap and average transmittance) with a spatial resolution of 1 mm, Stamate was able to demonstrate that the resistivity could vary by more than two orders of magnitude over a span of only 5 mm on the sample surface when using a 2-inch target [27]. This fact was proposed as a possibility to use the information embedded in the spatially distributed profiles for understanding the direct role of negative ions in the thin film growth mechanism. For example, attempts to investigate AZO properties by rotating the substrate or by neglecting the importance of the location on the sample where the thin film properties have been measured can lead to questionable results. Early on, several solutions to reduce the detrimental role of negative ions have been proposed [28], including no direct sight of the target by the substrate or reduction in the bias voltage [26,29], while very recently, Stamate demonstrated that it was possible to reduce the oxygen negative ion energy by reducing the acceleration bias over the sheet (DC self-bias) using a tuning electrode [30]. Consequently, a very narrow range of pressure was identified for obtaining a lower and more uniformly distributed resistivity over the sample surface. Moreover, a detailed investigation of plasma parameters by a dual thermal-electrostatic probe revealed that the narrow pressure range corresponded to the transition from a plume-like discharge to a magnetron discharge [31]. 

Metal oxide thin films deposited by magnetron sputtering are extensively used for a very large number of applications including thin film transistors [32,33,34,35], gas sensors [36], MEMS [37] and optoelectronic applications [38,39,40,41]. Moreover, many doped zinc oxides are investigated as potential transparent conducting layers [1,39]. In this context, it is relevant to know to what extent it would be possible to use the tuning electrode for influencing the properties of other metal oxide thin films. 

The aim of this work is to make a comparative study between spatially resolved thin film properties of AZO, ITO and gallium-doped zinc oxide (GZO) to assess if the tuning electrode is also effective in reducing the energy of the oxygen negative ions by reducing the self-bias and, if possible, what influence this has on thin film optoelectronic properties. Additional post annealing and degradation tests are also used to assess the thin film response, depending on local parameters. 

## 2. Materials and Methods

**Figure 1:**
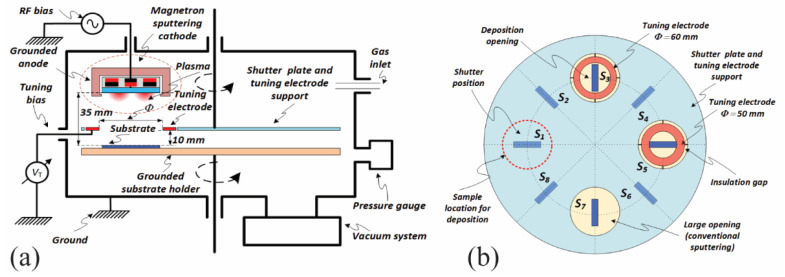
(**a**) Schematic of the experimental setup, (**b**) sample distribution on the substrate holder including the tuning electrodes.

The schematic diagram of the sputtering system used to deposit the AZO (2% Al_2_O_3_ to 98% ZnO by weight), GZO (5% Ga_2_O_3_ to 95% ZnO by weight) and ITO (10% SnO_2_ to 90% indium oxide) thin films on soda lime glass substrates (10 mm in width and 50 mm in length) is presented in Figure 1a. A balanced magnetron sputtering cathode (TORUS^®^ by Lesker) for 2-inch targets was placed on the top flange of a vacuum chamber. Up to eight samples could be symmetrically distributed on a grounded-substrate-holder that could expose each sample to the target by rotation. A large and rotatable plate was placed between target and substrate, and it could act as a shutter (during pre-sputtering) or interpose two different tuning electrodes with openings of 50 and 60 mm respectively (see Figure 1b). The opening of 100 mm was used to perform conventional deposition. The main advantage of this configuration is the ability to deposit consecutive samples without breaking the vacuum or even turning off the power supply while making small adjustments (with shutter on) for power, pressure and tuning electrodes. Following previous optimization [30], the target-to-substrate distance was kept constant at 35 mm and the tuning-electrode-to-substrate distance was kept constant at 10 mm. All films were deposited using the tuning electrode of a 60 mm diameter opening, electrically connected to ground. The RF power was 30 W for a deposition time of 30 min. Annealing was performed in a vacuum at the pressure of 7.5 Torr. The chamber was flushed with N_2_ gas to minimize the presence of oxygen during the process and was heated to 400 °C with a ramp-up rate of 24 °C/min and subsequently kept at 400 °C for 30 min following cooling down to room temperature. The accelerated degradation test was conducted on AZO and GZO for 100 s and 25 s, respectively, to identify the stability of the deposited materials upon exposure to a mild acetic acid (10% dilution in distilled water). The DC self-bias voltage was measured using the RF generator (300 W, Kurt Lesker, Jefferson Hills, PA, USA). The sheet resistance was measured both with a custom-made and a commercial system (KSR-4, Everbeing Int’l Corp., Hsinchu, Taiwan). The custom system consisted of two pads distributed perpendicular to the sample length for providing a uniform current and two pins for measuring the drop voltage. The film thickness was measured with a Filmetrics 20 instrument while the resistivity, carrier concentration and mobility were measured with a Nanomagnetics setup. The transmittance spectra were measured with a CRAIC 20/30 PV^TM^ microspectrometer with a spatial resolution of 1 mm. An X-ray Photoelectron Spectroscopy (XPS) Nexsa (Thermo Fisher Scientific, Waltham, MA, USA) with reflection electron energy loss spectroscopy (REELS) was used to measure the atomic composition and the bandgap.

## 3. Results and Discussion

### 3.1. Self-Bias and Main Thin Film Properties 

**Figure 2:**
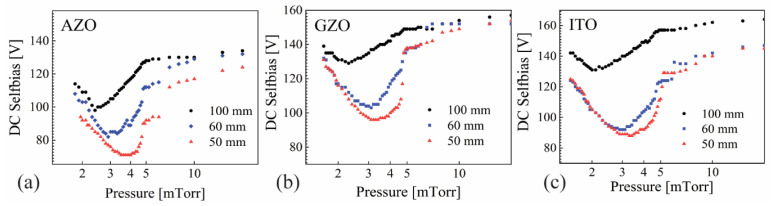
DC self-bias as a function of pressure for (**a**) AZO, (**b**) GZO and (**c**) ITO, with tuning electrodes of 50 mm and 60 mm, and the large opening of 100 mm.

The DC self-bias as a function of pressure for tuning electrodes of 50 mm and 60 mm in diameter and the large opening of 100 mm are presented in Figure 2a for AZO, Figure 2b for GZO and Figure 2c for ITO. Remarkably, all three materials exhibited the same behavior, with a fast decrease starting at 2 mTorr and a minimum value around 3.5 mTorr followed by a steep increase up to 5 mTorr and a steady but less accentuated increase for higher pressures. Moreover, the tuning electrode was able to lower the self-bias with more than 30 V for all three oxides near the minimum value, with slightly lower values when using the 50 mm opening compared to 60 mm. A certain degree of similitude was expected for AZO and GZO but not for ITO due to its much higher level of metal doping and the difference in both the base metal and dopant with respect to AZO.

As reported, the tuning electrode improves the electromagnetic coupling in the transition range from a plume-like discharge to magnetron discharge [30]. Oxygen negative ions can be produced not only in the proximity of the target but also in plasma volume, where low-energy electrons are produced by ionization [14,18]. 

However, such electrons gain less energy with respect to those accelerated in the plasma sheath, even though plasma potential was reported to be negative for discharge pressures below 5 mTorr. The influence of target-to-substrate distance and RF power on the self-bias was already investigated with the conclusion that low values can only be obtained for low powers (below 30 W for 2-inch targets) and short distances [27]. 

**Figure 3:**
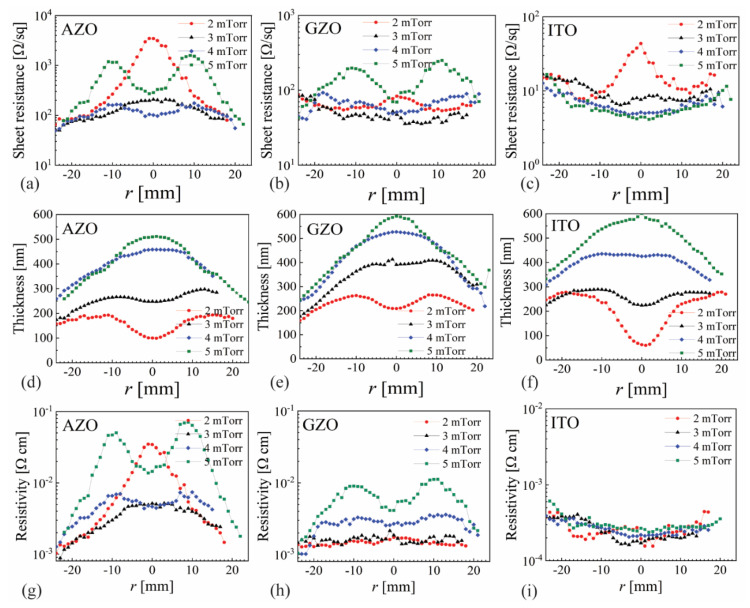
The spatial distribution of sheet resistance, film thickness and resistivity for (**a**,**d**,**g**) AZO, (**b**,**e**,**h**) GZO and (**c**,**f**,**i**) ITO.

To assess the possible influence of self-bias on thin film properties, samples have been deposited for each material at the four most-relevant pressures: 2, 3, 4 and 5 mTorr. To avoid shadowing effects very close to the substrate edge, all samples have been deposited using the 60 mm opening. The spatial distribution for sheet resistance, film thickness and resistivity are presented in Figure 3a,d,g, respectively, for AZO; Figure 3b,e,h, respectively, for GZO; and Figure 3c,f,i, respectively, for ITO. Similar results have already been reported for AZO [27,30], which facilitates summarizing them in direct correlation with GZO and ITO. At 2 mTorr, the discharge is in plume-like mode [31], with significant re-sputtering that reduces the film thickness for all materials at the substrate center (−10 < *r* < 10 mm). Preferential Zn re-sputtering over Al caused AZO sheet resistance values to be more than an order of magnitude higher at the center with respect to the sample edge, as can be seen in Figure 3a. 

**Figure 4:**
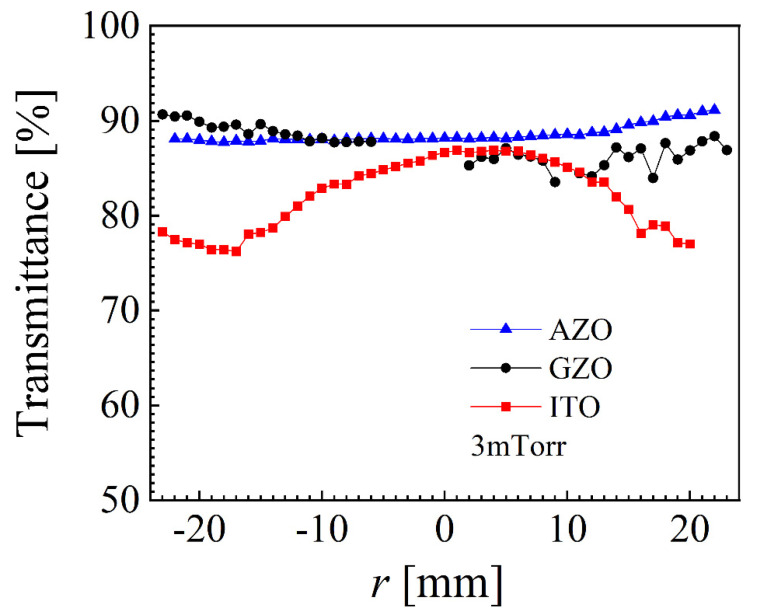
Average transmittance for AZO, GZO and ITO at 3 mTorr.

While re-sputtering was also present for GZO and ITO, it looks like ITO was more sensitive than GZO both in thin film decrease and higher sheet resistance values for −10 < *r* < 10 mm at 2 mTorr. In terms of resistivity, ITO shows very flat values for all pressures with slightly higher values towards the sample edge. A certain distribution was present for GZO, with higher values correlated with the erosion track only for 5 mTorr. AZO exhibited the most notable pressure dependence, as already reported [27], with high values at the sample center for 2 mTorr and two peaks correlated with the erosion track at 5 mTorr. ITO clearly overperforms AZO and GZO in resistivity, with almost 10 times lower values. The pressure of 3 mTorr resulted in the lowest resistivity for all three materials, and consequently, the samples deposited at this value were used to measure the average transmittance with a spatial resolution of 1 mm, as presented in Figure 4. While AZO exhibited a very flat transmittance profile, with values above 88%, and GZO was also reasonably flat with values above 83%, ITO presented a clear parabolic dependence, with values below 80% towards the sample edge. Smaller samples of 10 × 10 mm^2^ were cut from the whole sample of 10 × 50 mm^2^ at locations exhibiting the lowest resistivity, as follows: −25 ≤ *r* ≤ −15 mm for AZO and GZT and 0 ≤ *r* ≤ 10 mm for ITO. Such samples were used to measure the carrier concentration and mobility by the Hall effect, values that are presented in Table 1 alongside other relevant parameters. 

In terms of optoelectronic performance, AZO and GZO exhibit very close parameters with comparable values for resistivity, carrier concentration, mobility and transmittance. However, Ga is scarce and, consequently, a rather expensive material, suggesting that AZO is the better choice for large area applications. 

### 3.2. Post Annealing 

Post annealing is often used to improve the optoelectronic properties of TCOs even for thin films deposited with substrate heating [42,43,44,45]. Moreover, additional thermal processes are common for solar cells and other devices such as smart windows [46]. When used for low-emissivity windows [47], the TCO layer can also be exposed to glass tempering. Various reports presented the effect of substrate temperature during the deposition as well as the effect of thermal annealing on AZO [48,49,50,51,52,53], GZO [54,55,56,57] and ITO [58,59,60,61,62] properties. However, most of such studies concern small samples located at a certain position with respect to the target or larger samples that have been deposited under rotation. 

**Figure 5:**
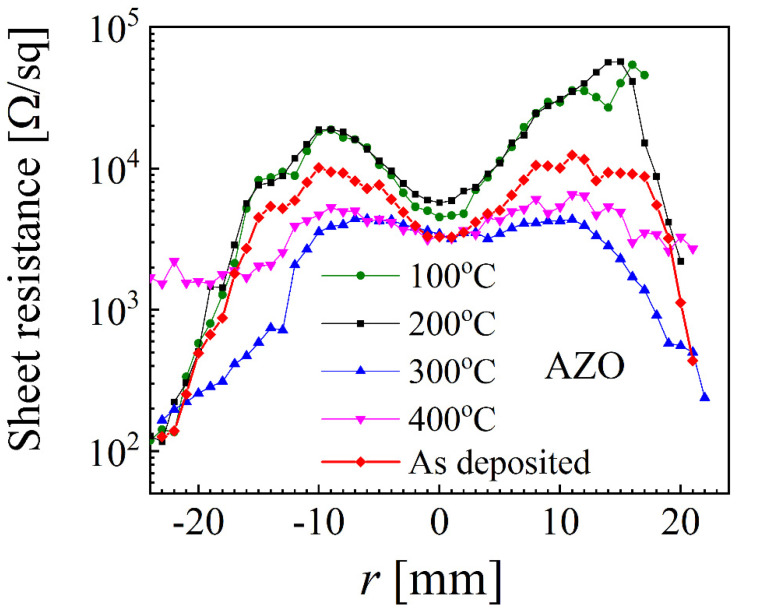
Spatial distribution of sheet resistance before and after different annealing temperatures over 30 min for AZO samples at 5 mTorr.

Consequently, it is important to know the annealing effect on the spatial distribution of the sheet resistance. Four AZO samples, deposited for similar parameters at 5 mTorr, were annealed at different temperatures for 30 min in vacuum. The sheet resistance values measured after the annealing are plotted together with the values of one sample prior to annealing, in Figure 5. As one can see, the change in sheet resistance is strongly dependent not only on the annealing temperature but also on the location. For *r* = 0 mm, the sheet resistance increased by a factor of 2 at 200 °C but remained unchanged for 300 °C and 400 °C. However, 200 °C and 300 °C resulted in one order of magnitude difference for locations correlated with the erosion track (*r* ≈ ±15 mm). Lower temperatures such as 100 °C and 200 °C are not able to propagate significant grain changes but can promote preferential diffusion towards the surface, leading to an observed increase in sheet resistance. However, much higher temperatures (300 °C and 400 °C) can increase the grain size and consequently promote a sheet resistance decrease by affecting the gain boundaries. Such aspects correlate very well with the already reported spatial distribution of the grain size that is expected to change by annealing and influence the sheet resistance [27,63]. 

Since 400 °C produced the most uniform sheet resistance for AZO, this value was used to anneal a new set of AZO, GZO and ITO samples deposited at 5 mTorr.

**Figure 6:**
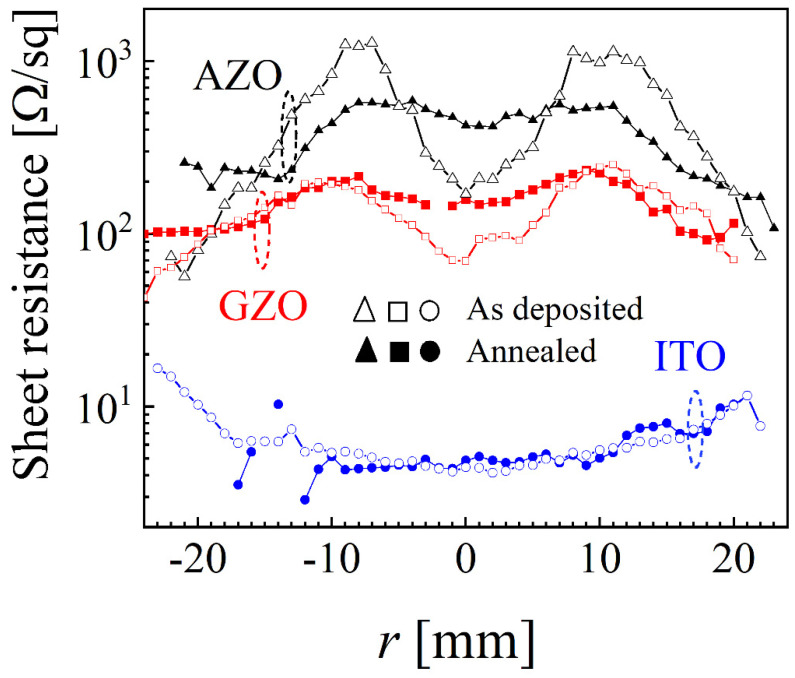
Spatial distribution of sheet resistance before and after annealing at 400 °C for AZO, GZO and ITO at 5 mTorr.

The spatial distribution for as-deposited and annealed samples for AZO, GZO and ITO is presented in Figure 6. While AZO presented significant variations (with higher values for −5 < *r* < 5 mm and |*r*| > 20 mm and lower values at locations correlated with the erosion track) and GZO noticeable differences (mainly for −10 < *r* < 10 mm), the annealing had almost no effect on the sheet resistance values for ITO. Overall, the annealing flattened the sheet resistance profile for both AZO and GZO, and this result once more emphasizes the importance of sample location and its size with respect to the target when investigating possible changes of electrical properties. The high stability of ITO for temperatures up to 400 °C was previously reported and is now reconfirmed [60].

### 3.3. XPS and REELS

**Figure 7:**
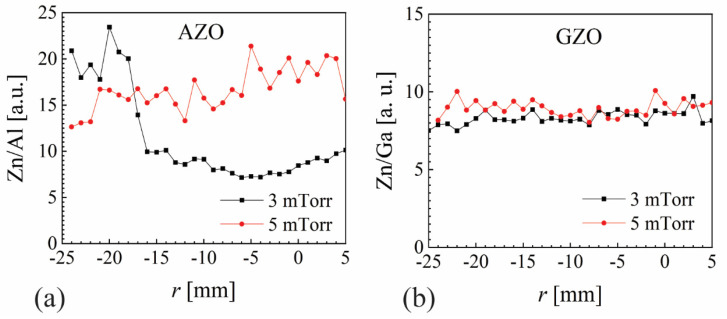
(**a**) Zn/Al and (**b**) Zn/Ga peak intensity ratios by XPS for films deposited at 3 and 5 mTorr.

Following the very distinct spatial-distribution profiles presented in Figure 3, one would expect to see them reflected in the analytical characterization by XPS, particularly for AZO and GZO at 5 mTorr. The Zn/Al and Zn/Ga intensity peaks are presented for 3 and 5 mTorr in Figure 7a,b, respectively. A high depletion of Zn is observed for AZO at 3 mTorr for |*r*| < 15 mm, a fact that can be associated with preferential Zn re-sputtering by energetic ions. However, no correlation with resistivity (see Figure 3g) is revealed for 5 mTorr (Zn/Al~15). The Zn/Ga ratio was very flat and almost independent of pressure, also in contrast with resistivity values at 5 mTorr (see Figure 3h) that presents one order of magnitude difference between values at |*r*|~10 mm and |*r*|~23 mm. The O-1s peak is often deconvoluted in three contributions associated with oxygen vacancies [48,64,65]. However, such a deconvolution (spatially distributed) presented only flat profiles, with no visible correlation with resistivity values.

**Figure 8:**
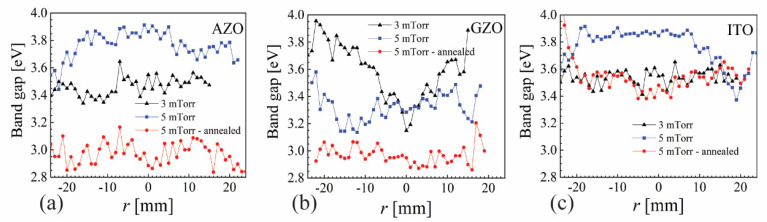
Bandgap for (**a**) AZO, (**b**) GZO and (**c**) ITO measured by REELS at 3 mTorr without annealing and at 5 mTorr before and after annealing at 400 °C.

Bandgap is an important parameter for TCOs and it was measured by REELS for AZO, GZO and ITO at 3 mTorr without annealing and at 5 mTorr before and after the annealing at 400 °C. The results are presented in Figure 8 and show a significant decrease in bandgap after annealing (5 mTorr), with a difference of about 0.8 eV for AZO and 0.3 for GZO and ITO. However, there is no noticeable correlation with resistivity values for AZO and GZO. The bandgap for GZO at 3 mTorr exhibits significantly lower values at the sample center with respect to the edge (about 0.8 eV higher); however, the resistivity is quite flat over the whole sample. This points to the need for additional analytical characterization methods in understanding the observed resistivity profiles. 

### 3.4. Wet Etching

**Figure 9:**
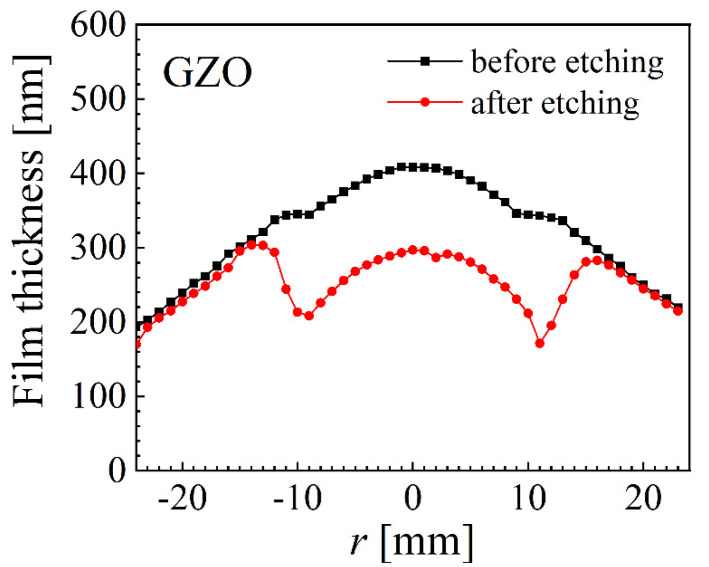
Film thickness before and after 25 of wet etching for an GZO sample deposited at 5 mTorr.

The degradation of samples deposited at 5 mTorr was carried out by immersion in a 10% solution of acetic acid. The wet etching was carried out after a preliminary etching rate was established with dummy samples. After this test, the AZO was immersed in solution for 100 s and GZO for 25 s, respectively. These times were selected to approximately result in a similar amount of removed material from both samples. However, this was not the case with the whole sample immersed, mainly due to variations in the etching rate of the sample at different positions, depending on the crystal structure of the sample surface, as can be seen from Figure 9, where the film thickness before and after the wet etching is presented. While AZO thickness was significantly reduced all over the sample, with a slight but noticeable difference correlated with the erosion track, the GZO exhibited a negligible thickness reduction for |*r*| > 15 mm and a significant reduction for the rest of the sample, with two clear valleys at the erosion track. 

**Figure 10:**
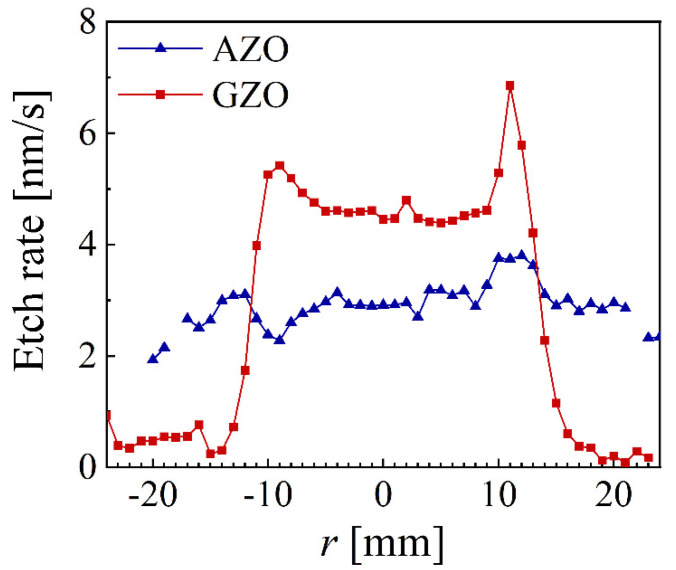
The spatial distribution of the etching rate for AZO and GZO samples deposited at 5 mTorr.

The calculated etching rate is presented in Figure 10 for both AZO and GZO, with about 3 nm/s all over the surface for AZO and extremely large differences for GZO, reaching above 4.5 for |*r*| < 12 mm and below 0.2 nm/s for |*r*| > 15 mm. The ITO samples exhibited no thickness reduction even for an immersion time of 5 min.

## 4. Conclusions

The known ability of the tuning electrode to reduce the self-bias with more than 25 V for AZO was also confirmed for GZO and ITO. The pressure range for this reduction (2 to 5 mTorr) was similar for all three materials. However, noticeable differences were observed within this pressure range. While significant re-sputtering was observed at 2 mTorr for all three metal oxides, only AZO and ITO exhibited significantly higher resistivity values at the sample center. Most uniform resistivities were obtained at 3 and 4 mTorr, while 5 mTorr presented an accentuated erosion track dependence for AZO, a mild dependence for GZO and no influence at all for ITO. While the resistivity of ITO was almost 10 times lower than that of AZO and GZO, its transmittance was not as good, with values exhibiting values with 10% lower near the edge of the sample. The annealing effect was strongly dependent on the location over the sample with respect to the erosion track for AZO and GZO, while ITO was almost insensitive to annealing for temperatures below 400 °C. Analytical characterization by XPS revealed no direct correlation of the atomical composition and the bandgap with the erosion track for the AZO and GZO samples deposited at 5 mTorr, in contrast with resistivity values. Pressure and annealing induced large changes in bandgap values for all three metal oxides. However, previously reported bandgap values for AZO measured from Tauc’s plot presented a clear correlation with the erosion track [27], a fact that needs to be further investigated. Wet etching by diluted acetic acid revealed a high and rather uniform etching rate of 3 nm/s for AZO, while the etching rate was very strongly correlated with the erosion track for GZO, with almost no thickness reduction at the sample edge. The included results re-emphasize the ability of the spatially resolved parameters for metal oxides deposited by sputtering to reveal valuable information that links the sputtering target, the anisotropic magnetic field and the three-dimensional distribution of plasma with the properties of the film deposited on the substrate. 

## Figures and Tables

**Table 1:** Optoelectronic parameters obtained at locations exhibiting the lowest resistivity.

Material	Sheet Resistance (Ω/sq)	Thickness (nm)	Resistivity (Ω cm)	Carrier Concentration (cm^−3^)	Hall Mobility (cm^2^/Vs)	Transmittance (%)
AZO	50.7	230	1.16 × 10^−3^	4.8 × 10^20^	11.1	88%
GZO	81.8	190	1.5 × 10^−3^	4.5 × 10^20^	8.9	90%
ITO	9.4	262	2.4 × 10^−4^	7.8 × 10^20^	32.4	86%

## Data Availability

The data can be made available from the corresponding author upon request.
